# Determinants of health status and health-related behaviors among polish firefighters: a nationwide survey-based study

**DOI:** 10.3389/fnut.2025.1682811

**Published:** 2025-11-28

**Authors:** Łukasz Dudziński, Jan Tymiński, Tomasz Kubiak, Lukasz Czyzewski, Janusz Wyzgal, Robert Gałązkowski

**Affiliations:** 1 Department of Medical Rescue, Medical University of Warsaw, Warsaw, Poland; 2 Department of Medical Rescue, Fire University, Warsaw, Poland; 3 Department of Health Sciences, Poznan Medical Academy of Applied Sciences Mieszko I, Poznań, Poland; 4 Department of Geriatric Nursing, Medical University of Warsaw, Warsaw, Poland; 5 Department of Nephrologic Nursing, Medical University of Warsaw, Warsaw, Poland

**Keywords:** occupational health, BMI, firefighter, nutrition, physical activity

## Abstract

**Objective:**

To assess health practices among Polish firefighters in relation to the high physical and mental demands of their profession.

**Materials and methods:**

A cross-sectional survey design with categorical data analysis was used. An online survey was conducted between 10 October 2024 and 25 October 2024. The survey covered 856 officers serving in organisational units of the State Fire Service (SFS) throughout Poland. The survey was based on a proprietary questionnaire, including demographics and individual health practices: nutrition, stimulants, sleep, physical activity.

**Results:**

Eight hundred seventeen respondents who answered all questions were included in the analysis. The analysis of the results did not show any statistically significant differences between the genders in terms of: participation in sports/physical activity (yes vs. no) (*p* = 0.088); frequency of physical activity (*p* = 0.541); type of diet (*p* = 0.741). Persons employed in command positions were the least likely to smoke cigarettes (*p* < 0.001). These people most often indicated that they were very satisfied with their health (*p* < 0.001). People in command positions were more likely to declare unhealthy eating habits, but at the same time they assessed their health better.

**Conclusion:**

This nationwide survey identified clear, actionable patterns in firefighters’ health behaviors. Overweight and obesity were common, and multiple behaviors clustered with higher BMI. Prevention should be targeted across career stages and supported organizationally integrating nutrition, hydration, sleep hygiene, and physical training with enabling conditions (duty scheduling, healthy food/water access on shift, protected time and facilities for exercise). Future studies should include objective measurements, validated psychosocial scales, and longitudinal designs to clarify directionality and evaluate interventions.

## Introduction

Polish firefighters are a professional, uniformed and specially equipped formation whose main task is to fight fires, natural disasters and other local hazards. The fire brigade carries out rescue operations in various specialised areas classified as specialist rescue: technical, high-altitude, water and diving, chemical and ecological rescue. The scope of firefighters’ activities is extensive, which is associated with a wide range of hazards. Firefighters are exposed to adverse conditions and factors that may directly or indirectly affect their health or life (1–3).

Some harmful factors are non-modifiable or difficult to modify. This group includes the risk related to exposure to chemical agents such as toxic combustion products, where contact can be limited but is difficult to eliminate completely (4, 5).

Other occupational risks stem from the effects of stress. Firefighters experience chronic stress linked to organizational culture and the tension of waiting for a call, as well as acute stress, including traumatic stress, resulting from interactions with people in life-threatening emergencies. This stress increases the risk of somatic diseases and mental disorders and raises the risk of death by suicide (6–8). These issues are recognized, and a range of preventive and prophylactic measures are being implemented worldwide (9, 10).

The Polish fire service system is divided into shift work (24 h) and daily work (8 h). However, interventions (rescue and firefighting operations) involve a change to unfavorable conditions, including fires, road, rail or air accidents, operations in water areas, at height, natural, industrial or construction disasters (11, 12).

According to the occupational hazard characteristics sheet, there are many occupational hazards in the working environment of firefighters (in the working environment and in the way the work is performed), which have an impact on health (13, 14).

The profession of a firefighter involves contact with people in danger, exposure to traumatic events, and working under time pressure, which is associated with mental stress (15–17).

Health risks in the fire service are associated with duties other than interventions. Furthermore, there are factors that pose a risk in the long term: air pollution, heat stress, extreme physical exertion, mental stress and altered circadian rhythms. All these factors can contribute to both short-term and long-term deterioration of the physical and mental health of firefighters (18–20).

A systemic solution for health monitoring consists of annual periodic examinations and physical fitness assessments, as well as psychologists employed by provincial fire service headquarters to look after the mental health of firefighters (21–23). Numerous studies and scientific projects are also being carried out on the ergonomics of work, equipment, control and improvement of the working environment (24–26).

Even with these solutions in place, growing attention is being paid to lifestyle and daily health practices, such as physical activity, nutrition, sleep quality, hydration and maintaining a healthy body weight. However, it is important that firefighters themselves are aware of and take an individual approach to health checks. A proper diet, physical activity, maintaining a healthy body mass index (BMI), check-ups and participation in age-appropriate preventive programmes are all important. Many studies point to diseases occurring after many years of service or after retirement. Fire pollutants are associated with chronic late health effects such as cancer. Carcinogenic substances are identified on firefighters’ personal protective equipment, equipment and fire station surfaces (27, 28).

Although the evidence base on occupational risks and illnesses among firefighters is expanding, relatively little is known about the daily health practices of State Fire Service (SFS) officers, such as diet, sleep, physical activity levels, and the use of stimulants and dietary supplements. Furthermore, the literature on the subject insufficiently describes how these practices vary depending on age, position, length of service, and education. Understanding these relationships can provide a foundation for planning effective preventive measures that take into account the career stage and individual needs of officers. However, up-to-date nationwide data on daily health practices in Poland remain scarce, especially when stratified by age, length of service, position, and education. To date, studies have focused mainly on single lifestyle components (for example physical activity or nutrition) in local or specialty samples, and they rarely examine the co-occurrence of habits (diet, sleep, activity) or their variation across the career cycle. There is also a lack of consistent, operational definitions of health behavior indicators tailored to shift work realities (for example meal and sleep regularity), and results for Polish firefighters are scattered and not calibrated to the position structure (29–33).

This gap limits the design of targeted nutritional and behavioral interventions across the career. It also hinders the identification of intervention windows (entry into service, years of service, promotion to command roles) when habit profiles may be most modifiable.

Research questions:

1. What are the prevalence and patterns of diet, sleep, physical activity, and the use of stimulants and dietary supplements among SFS officers?
2. What are the associations between BMI and physical activity, sleep, and dietary patterns?
3. How do age, length of service, and position differentiate these practices?
4. Do combinations of habits (for example low activity with irregular meals and shorter sleep) form identifiable clusters of behaviors linked to higher BMI?

We prioritize an integrated approach that considers co-occurrence and potential interactions between health habits and the characteristics of service. The current literature indicates rapid shifts in operational exposures and health culture within rescue formations. There is a need to update nationwide data for professional firefighters in Poland to guide interventions at recruitment, training, and promotion stages. We therefore formulated the following hypotheses:

Greater length of service and command positions will be associated with less favorable dietary patterns and lower activity. Higher BMI may be associated with lower physical activity and poorer sleep hygiene.

## Objective

The aim of the work was to evaluate selected health practices among Polish firefighters in relation to their occupation, which involves significant physical and mental stress.

## Materials and methods

### Research design

We conducted a nationwide, cross-sectional online survey of SFS personnel in Poland between 10 and 25 October 2024. The target population comprised officers from all 16 voivodeships serving in diverse organisational settings: Rescue and Firefighting Units (24-h shift system), municipal/district and provincial headquarters (8-h daily system), as well as training centers and the national firefighting academy. Recruitment was carried out via official internal channels (intranet, distribution by line managers, mailing lists). Participation was voluntary and anonymous; each invited officer could submit one unique response using Google Forms on service or private devices.

The proprietary 19-item questionnaire consisted of two sections: Part A (demographics) and Part B (lifestyle: nutrition, physical activity, sleep, stimulants, hydration). The SFS operational context, high physical and psychological loads, shift work, heat and contaminant exposure, and mandatory periodic medical and fitness assessment-justifies examining everyday health practices as factors relevant to operational readiness. Findings pertain to the professional SFS and are not intended to be directly generalised to volunteer fire brigades or other uniformed services. A total of 856 questionnaires were received; details of the analytical sample are reported in the Results.

### Variables

Dependent (outcome) variables: (1) BMI category calculated from self-reported height/weight and classified into mutually exclusive WHO bands: <18.5 (underweight), 18.5–24.9 (normal weight), 25.0–29.9 (overweight), ≥30.0 (obesity); for selected comparisons we additionally used binary contrasts (≥25.0 vs. 18.5–24.9; ≥30.0 vs. 18.5–24.9); (2) physical activity frequency: less than once/week, 1–2, 3–4, 5–6, every day (with a pre-specified sensitivity contrast ≤2 vs. ≥ 3 sessions/week); (3) sleep duration: <6, 6, 7, 8, 9, >9 h; (4) meal pattern: regularity (yes/no) and number of meals/day (1, 2, 3, 4, >4); (5) dominant cooking method: boiling, steaming, frying, roasting, grilling, other; (6) hydration: 0.5, 1, 1.5, 2, >2 L/day; (7) smoking status/duration: non-smoker = no nicotine use (combustible tobacco, nicotine e-cigarettes and other nicotine products excluded; nicotine-free e-cigarettes counted as non-smoker); duration among smokers: <5, 6–10, 11–15, >15 years; (8) supplementation: categories of supplements (e.g., vitamin D, B vitamins, fish oils, folates, other) and source of recommendation (medical advice, own experience, scientific sources, family advice, advertising, other); (9) self-rated health: very dissatisfied, dissatisfied, do not know, satisfied, very satisfied. For multi-response items (cooking method, supplementation), each option was additionally coded as a binary indicator (0/1); if a single option was selected, it served as the dominant category in crosstabs.

Independent (predictor) variables: sex (male/female), age group (≤20; 21–30; 31–40; 41–50; 51–60 years), position (candidate/preparatory; rescuer in shifts; commander—shift; commander—daily; other daily; manager—daily), length of service (candidate; 3–9; 10–19; 20–29; ≥30 years), education (primary; secondary; higher—engineering; higher—master’s; higher—PhD).

### Operational definitions and measurement

Diet/meal pattern. “Type of diet” referred to the respondent’s dominant dietary pattern selected from a predefined list (“customary, daily”; “predominantly carbohydrates/protein/fat”; “predominantly plant products”; “collective feeding”; “other”). “Regularity of meals” was defined as eating at relatively fixed times on ≥5 days/week (yes/no). “Number of meals/day” captured the typical daily count (1, 2, 3, 4, > 4) within the reference period (see below). The “dominant cooking method” denoted the most frequently used heat-treatment method for main meals; where multiple options were ticked, additional indications were secondarily coded as binary (0/1) for descriptive tables. Physical activity. One “session” was defined *a priori* as ≥30 min of continuous activity at least of moderate intensity (perceived faster breathing and/or heart rate) or an equivalent intermittent effort. Frequency categories summarised the typical number of such sessions per week; the sensitivity contrast ≤2 vs. ≥ 3 sessions/week was pre-specified. Sleep. “Sleep duration” pertained to the average length of the principal nocturnal sleep (naps excluded), aggregated to the nearest category across duty and off-duty days. Hydration. “Fluid intake per day” quantified self-reported total volume of non-alcoholic beverages consumed on a typical day (water, tea, coffee, isotonic drinks, juices), with categories 0.5, 1, 1.5, 2, >2 L/day; alcohol was explicitly excluded from hydration metrics. Stimulants and supplementation. “Smoking status/duration” categories followed the questionnaire (non-smoker, <5, 6–10, 11–15, >15 years; e-cigarettes included within non-smoker only if completely nicotine-free). Supplementation was a multi-response item (e.g., vitamin D, B vitamins, fish oils, folates, other) with parallel binary coding (0/1) and a single item on the primary source of recommendation (medical advice, own experience, scientific sources, family advice, advertising, other).

### Data analysis

In order to determine the relationship between the characteristics of the study group (gender, age, BMI, position, seniority, education) and the answers to questions determining the respondents’ attitude toward a healthy lifestyle, Pearson’s chi-square tests were performed. A test probability level of *α* = 0.05 was applied. In the case of statistically significant differences (*p* < 0.05), Cramer’s Fi or V correlation coefficients were also determined (depending on the number of levels of the characteristics studied). Missing data were handled under a Missing Completely at Random (MCAR) assumption using complete-case analysis (CCA). Of the 856 submitted questionnaires, 817 met the complete-case definition and entered the analytical dataset (39 exclusions; 4.6%). Given the low overall missingness and the predominance of mandatory items in the online form, MCAR was deemed a reasonable working assumption for CCA. No single or multiple imputation was performed. Descriptive statistics and statistical tests were performed using Statistica 13.3 (TIBCO Software Inc.).

### Research setting

The study was based on a proprietary questionnaire. The data included:

- demographics—six variables categorising respondents: Gender, Age, BMI, Job title, Length of service, Education,
- questions about lifestyle—Eating habits, type of diet, physical activity, stimulants, hydration, sleep.
- The survey questionnaire consists of questions:
- with one possible answer (11 questions),
- with multiple answers possible (4 questions),
- based on a 5-point Likert scale (2 questions),
- YES/NO questions (2 questions).

Inclusion criteria comprised (1) active service in the SFS at the time of survey administration, (2) age ≥18 years, (3) informed consent, and (4) submission of a unique response (survey access limited to one submission per user). Exclusion criteria were non-active status, declined consent, duplicate/technically corrupted entries, and incomplete core data. Plausibility and consistency checks were specified *a priori* (e.g., non-negative durations, mutually exclusive single-choice items, anthropometry within humanly plausible ranges); entries failing these checks were set to missing at the item level and excluded from complete-case analyses.

### Pilot testing and content evaluation

The questionnaire was piloted with 25 firefighters from three Rescue and Firefighting Units representing different regions of the country and a range of positions and duty systems (5–10 September 2024). Pilot participants were not included in the main sample. The pilot assessed question clarity, the adequacy of operational wording, and completion time across diverse groups (different positions and duty systems). No systematic ambiguities or response-mapping issues were noted. The mean completion time fell within the planned range, and the distribution of answers ensured satisfactory variability. As a result, no substantive or editorial changes were required before distributing the final version. The questionnaire received a positive content validity assessment in expert review (alignment of items with intended constructs and service realities) and showed good acceptability in the pilot (readability, completion time, no trap items). A full psychometric validation was not conducted (for example reliability analyses for multi-item scales or test- retest). We recognize this as a limitation and indicate it as a direction for further tool development.

### Ethical considerations

The survey was designed as a fully anonymous tool, and participation was voluntary, and respondents were informed of this. In June 2024, permission was obtained from the SFS Chief Commander to conduct a survey among serving firefighters, and permission was obtained from the rector of the Firefighting Academy to conduct a survey among cadets serving as candidates as part of their uniformed studies. The actual survey was preceded by a pilot study conducted on 10–15 September 2024 at three randomly selected SFS’ Rescue and Firefighting Unit from three different regions of the country (Lubelskie, Warmińsko-Mazurskie and Zachodniopomorskie voivodeships/provinces). The aim of the pilot study was to assess whether the content of the questionnaire was clear and understandable. Due to the lack of comments during the pilot study, no corrections were made to the questionnaire before its full distribution. The analysis complies with the principles of the Helsinki Declaration. For the purposes of the project, on 23 October 2023, a positive opinion was obtained from the Bioethics Committee of the (John Paul II University, Poland) - decision no. 12/2023.

## Results

The study included 817 respondents who answered all questions. Questionnaires that were incomplete were rejected at the stage of preparing the database for statistical calculations. Among the respondents, there were 748 men (92%) and 69 women (8%). The results are presented in Table 1.

**Table 1:** General characteristics of the study group.

Age	*N* (%)
to 20 years	41 (5)
21–30 years	207 (25)
31–40 years	313 (38)
41–50 years	231 (28)
51–60 years	25 (3)
BMI, N (%)	
<18.5	3 (<1)
18.5–24.9	339 (41)
≥25	388 (47)
≥30	87 (11)
Position, N (%)	
Candidate/preparatory service	133 (16)
A rescuer in shifts	253 (31)
Commander in a shift system	140 (17)
Commander in a daily system	125 (15)
Other positions in daily system	98 (12)
Manager in daily system	68 (8)
Seniority, N (%)	
Candidate/preparatory service	169 (21)
3–9 years	148 (18)
10–19 years	339 (41)
20–29 years	110 (13)
30 years and more	51 (6)
Education, N (%)	
Primary	2 (<1)
Secondary	322 (39)
Higher: Engineering degree	270 (33)
Higher: Master’s degree	210 (26)
Higer: PhD	13 (2)

The analysis of the results did not show any statistically significant differences between the genders in terms of: participation in sports/physical activity (yes vs. no) (*p* = 0.088); frequency of physical activity (0.541); type of diet (*p* = 0.741); number of meals consumed per day (*p* = 0.052); regularity of meals (*p* = 0.119); frequency of paying attention to the composition of meals (*p* = 0.247); smoking (*p* = 0.327); length of sleep (*p* = 0.061). However, statistically significant differences were found for gender in relation to: frying, where men *N* = 225 (30%) vs. women *N* = 9 (13%), *p* = 0.003; number of liters of fluids consumed per day (*p* = 0.010); taking dietary supplements, where women more often used B vitamins *N* = 70 (9%) vs. *N* = 13 (19%) *p* = 0.013, and the reason for taking supplements, i.e., where women more often took them on the advice of a doctor *N* = 51 (7%) vs. *N* = 15 (22%) *p* = 0.001.

The analysis of the results did not show any statistically significant differences in BMI in relation to: the number of meals consumed per day (*p* = 0.289); the method of meal preparation (*p* = 0.750). However, statistically significant differences were found for BMI in relation to declared physical activity (*p* < 0.001), where people who did not declare physical activity had significantly higher BMI values. A similar correlation was also found for the frequency of physical activity (*p* < 0.001). A correlation was found between not following a diet (*p* = 0.008), irregular meal times (*p* = 0.002) and reduced sleep time (*p* = 0.034) and an increase in BMI. In the case of cigarette smoking, an inverse relationship was found, i.e., people who smoked tobacco or e-cigarettes had statistically significantly lower BMI values (*p* < 0.001). Overweight and obese people who took dietary supplements did so more often on the recommendation of a doctor (*p* = 0.001).

In a comparative analysis of positions and length of service, clear nutritional trends were observed. It was shown that length of service had a statistically significant effect on the type of diet followed, i.e., people with longer service were less likely to follow a customary and daily diet (*p* < 0.001). A similar correlation was found for job position (Table 2). It was shown that people employed in command positions statistically significantly more often prepared fried dishes (*p* < 0.001) and least often used boiling (*p* < 0.001). These individuals consumed the lowest amount of fluids per day (*p* < 0.001).

**Table 2:** Comparative analysis of nutritional factors, position and length of service.

Variable	Seniority						Position						
	Candidate/preparatory service *N* = 169 (21%)	3–9 years *N* = 148 (18%)	10–19 years *N* = 339 (41%)	20–29 years *N* = 110 (13%)	30 years and more *N* = 51 (6%)	*p*	Candidate/preparatory service *N* = 133 (16%)	Rescuer in shifts *N* = 253 (31%)	Commander in a shift system *N* = 140 (17%)	Commander in a daily system *N* = 125 (15%)	Other positions in daily system *N* = 98 (12%)	Manager in daily system *N* = 68 (8%)	*p*
Type of diet N (%)													
Customary, daily	117 (69)	102 (69)	205 (60)	75 (68)	10 (20)	<0.001 (0.242)	89 (67)	172 (68)	91 (65)	31 (25)	74 (76)	52 (76)	<0.001 (0.337)
Predominantly carbohydrates	8 (5)	10 (7)	10 (3)	5 (5)	1 (2)	0.337	7 (5)	16 (6)	6 (4)	1 (1)	1 (1)	3 (4)	0.096
Predominantly protein-rich	24 (14)	24 (16)	44 (13)	14 (13)	0 (0)	0.056	17 (13)	47 (19)	20 (14)	6 (5)	10 (10)	6 (9)	0.006 (0.141)
Predominantly fat-rich	1 (1)	3 (2)	53 (16)	2 (2)	0 (0)	<0.001 (0.275)	1 (1)	5 (2)	14 (10)	37 (30)	1 (1)	1 (1)	<0.001 (0.388)
Predominantly of plant products	0 (0)	3 (2)	17 (5)	5 (5)	1 (2)	0.029 (0.115)	0 (0)	4 (2)	4 (3)	10 (8)	6 (6)	2 (3)	0.002 (0.151)
Collective feeding	16 (9)	2 (1)	1 (<1)	0 (0)	0 (0)	<0.001 (0.244)	17 (13)	2 (1)	0 (0)	0 (0)	0 (0)	0 (0)	<0.001 (0.307)
Other	3 (2)	4 (3)	9 (3)	9 (8)	39 (76)	<0.001 (0.663)	2 (1)	7 (3)	5 (4)	40 (32)	6 (6)	4 (6)	<0.001 (0.386)
How many meals do you eat per day? N (%)													1
0 (0)	0 (0)	2 (1)	0 (0)	0 (0)	<0.001 (0.135)	0 (0)	0 (0)	1 (1)	0 (0)	0 (0)	1 (1)	<0.001 (0.176)	2
2 (1)	7 (5)	12 (3)	6 (5)	0 (0)		1 (1)	9 (3)	6 (4)	2 (1)	8 (8)	1 (1)		3
61 (36)	52 (35)	179 (53)	48 (44)	42 (82)		50 (38)	93 (37)	73 (52)	97 (78)	40 (41)	29 (43)		4
82 (49)	71 (48)	111 (33)	44 (40)	4 (8)		60 (45)	126 (50)	38 (27)	22 (18)	42 (43)	24 (35)		> 4
24 (14)	18 (12)	35 (10)	12 (11)	5 (10)		22 (16)	25 (10)	22 (16)	4 (3)	8 (8)	13 (19)		Do you eat regularly? *N* (%)
Yes	118 (70)	82 (55)	206 (61)	58 (53)	45 (88)	<0.001 (0.179)	97 (73)	143 (57)	74 (53)	106 (85)	48 (49)	41 (60)	<0.001 (0.247)
No	51 (30)	66 (45)	133 (39)	52 (47)	6 (12)		36 (27)	110 (43)	66 (47)	19 (15)	50 (51)	27 (40)	
How do you prefer to prepare hot meals? N (%)													Cooking
88 (52)	69 (47)	137 (40)	54 (49)	6 (12)	<0.001 (0.189)	66 (50)	121 (48)	68 (49)	20 (16)	49 (50)	30 (44)	<0.001 (0.236)	Steaming
13 (8)	15 (10)	26 (8)	5 (5)	3 (6)	0.551	11 (8)	25 (10)	10 (7)	2 (2)	8 (8)	6 (9)	0.126	Frying
44 (26)	37 (25)	86 (25)	26 (24)	41 (80)	<0.001 (0.296)	36 (27)	65 (26)	36 (26)	58 (46)	22 (22)	17 (25)	<0.001 (0.169)	Roasting
16 (9)	18 (12)	74 (22)	19 (17)	1 (2)	0.001 (0.170)	12 (9)	27 (11)	21 (15)	43 (34)	13 (13)	12 (18)	<0.001 (0.230)	Grilling
5 (3)	4 (3)	5 (1)	3 (3)	0 (0)	0.585	5 (4)	5 (2)	2 (1)	2 (2)	2 (2)	1 (1)	0.790	Other
3 (2)	5 (3)	11 (3)	3 (3)	0 (0)	0.624	3 (2)	10 (4)	3 (2)	0 (0)	4 (4)	2 (3)	0.307	Do you pay attention to the ingredients of your meals? *N* (%)
Never	17 (10)	10 (7)	28 (8)	4 (4)	3 (6)	0.002 (0.112)	14 (10)	26 (10)	7 (5)	3 (2)	7 (7)	5 (7)	0.005 (0.116)
Sometimes	64 (38)	54 (36)	131 (39)	55 (50)	11 (22)		49 (37)	103 (41)	61 (44)	30 (24)	41 (42)	31 (46)	
Often	69 (41)	65 (44)	141 (42)	38 (34)	20 (39)		56 (42)	94 (37)	53 (38)	70 (56)	34 (35)	26 (38)	
Always	19 (11)	19 (13)	39 (11)	13 (12)	17 (33)		14 (10)	30 (12)	19 (13)	22 (18)	16 (16)	6 (9)	
How much fluid you drink per day (in liters)? N (%)													0.5 L
1 (1)	3 (2)	10 (3)	2 (2)	0 (0)	<0.001 (0.125)	1 (1)	5 (2)	4 (3)	1 (1)	1 (1)	4 (6)	<0.001 (0.161)	1 L
15 (9)	16 (11)	35 (10)	15 (13)	4 (8)		10 (8)	21 (8)	11 (8)	6 (5)	22 (22)	15 (22)		1.5 L
49 (29)	41 (28)	112 (33)	47 (43)	12 (23)		39 (29)	70 (28)	41 (29)	64 (51)	27 (28)	20 (29)		2 l
50 (29)	42 (28)	99 (29)	24 (22)	33 (65)		39 (29)	80 (32)	44 (31)	46 (37)	23 (23)	16 (24)		>2 l
54 (32)	46 (31)	83 (25)	22 (20)	2 (4)		44 (33)	77 (30)	40 (29)	8 (6)	25 (26)	13 (19)		
													
													

Table 3 presents a comparative analysis of dietary supplements, job position and length of service. It shows, among other things, that people employed in command positions were more likely to take vitamin D supplements (*p* < 0.001). Table 4 presents a comparative analysis of physical activity, leisure time, use of stimulants, job position and length of service. People employed in command positions were the least likely to smoke cigarettes (*p* < 0.001). These people most often indicated that they were very satisfied with their health (*p* < 0.001).

**Table 3:** Comparative analysis of variables: dietary supplements used, position and length of service.

Variable	Seniority						Position						
	Candidate/preparatory service *N* = 169 (21%)	3–9 years *N* = 148 (18%)	10–19 years *N* = 339 (41%)	20–29 years *N* = 110 (13%)	30 years and more *N* = 51 (6%)	*p*	Candidate/preparatory service *N* = 133 (16%)	Rescuer in shifts *N* = 253 (31%)	Commander in a shift system *N* = 140 (17%)	Commander in a daily system *N* = 125 (15%)	Other positions in daily system *N* = 98 (12%)	Manager in daily system *N* = 68 (8%)	*p*
Dietary supplements (which ones)? N (%)													
Fish fats	26 (15)	16 (11)	40 (12)	14 (13)	2 (4)	0.262	22 (17)	29 (11)	14 (10)	10 (8)	15 (15)	18 (12)	0.96
Vitamin B	16 (9)	24 (16)	26 (8)	13 (12)	4 (8)	0.062	12 (9)	29 (11)	12 (9)	2 (2)	17 (17)	11 (16)	0.002 (0.154)
Folates	2 (1)	1 (1)	2 (1)	0 (0)	0 (0)	0.752	2 (2)	0 (0)	0 (0)	2 (2)	1 (1)	0 (0)	0.232
Vitamin D	20 (12)	29 (20)	88 (26)	18 (16)	4 (8)	0.0004 (0.158)	17 (13)	40 (16)	25 (18)	54 (43)	13 (13)	10 (15)	<0.0001 (0.258)
Vitamin K	0 (0)	0 (0)	7 (2)	0 (0)	0 (0)	0.041 (0.110)	0 (0)	1 (<1)	3 (2)	3 (2)	0 (0)	0 (0)	0.092
Vitamin A	1 (1)	1 (1)	1 (<1)	1 (1)	0 (0)	0.902	1 (1)	1 (<1)	1 (<1)	1 (1)	0 (0)	0 (0)	0.920
Other supplements	42 (25)	24 (16)	52 (15)	17 (15)	39 (76)	<0.0001 (0.360)	33 (25)	40 (16)	31 (22)	38 (30)	16 (16)	16 (24)	0.020 (0.128)
Do not use supplements	62 (37)	52 (35)	123 (36)	47 (43)	2 (4)	<0.0001 (0.175)	46 (35)	112 (44)	54 (39)	15 (12)	36 (37)	23 (34)	<0.0001 (0.220)
Why do you take dietary supplements? N (%)													
Source of knowledge (scientific literature)	19 (11)	31 (21)	31 (9)	18 (16)	0 (0)	<0.001 (0.167)	14 (11)	36 (14)	20 (14)	6 (5)	14 (14)	9 (13)	0.118
Family advice	16 (9)	13 (9)	18 (5)	3 (3)	3 (6)	0.129	14 (11)	17 (7)	9 (6)	4 (3)	6 (6)	3 (4)	0.275
Advertising on TV, press	2 (1)	2 (1)	12 (4)	4 (4)	0 (0)	0.240	2 (2)	6 (2)	3 (2)	8 (6)	1 (1)	0 (0)	0.046 (0.117)
Own experiences	69 (41)	38 (26)	138 (41)	35 (32)	5 (10)	<0.0001 (0.184)	59 (44)	77 (30)	49 (35)	48 (38)	29 (30)	23 (34)	0.095
Medical advices	13 (8)	18 (12)	20 (6)	10 (10)	5 (10)	0.208	10 (8)	18 (7)	7 (5)	5 (4)	16 (16)	10 (15)	0.003 (0.148)
Other	50 (30)	46 (31)	120 (35)	40 (36)	38 (75)	<0.0001 (0.214)	34 (26)	99 (39)	52 (37)	54 (43)	32 (33)	23 (34)	0.054

**Table 4:** Comparative analysis of physical activity, leisure, use of stimulants, position and length of service.

Variable	Seniority						Position						
	Candidate/preparatory service *N* = 169 (21%)	3–9 years *N* = 148 (18%)	10–19 years *N* = 339 (41%)	20–29 years *N* = 110 (13%)	30 years and more *N* = 51 (6%)	*p*	Candidate/preparatory service *N* = 133 (16%)	Rescuer in shifts *N* = 253 (31%)	Commander in a shift system *N* = 140 (17%)	Commander in a daily system *N* = 125 (15%)	Other positions in daily system *N* = 98 (12%)	Manager in daily system *N* = 68 (8%)	*p*
Do you practice sports or physical activity? N (%)													
Yes	159 (94)	137 (93)	293 (86)	80 (73)	47 (92)	<0.001 (0,203)	126 (95)	222 (88)	129 (92)	115 (92)	75 (77)	49 (72)	<0.001 (0.214)
No	10 (6)	11 (7)	46 (14)	30 (27)	4 (8)		7 (5)	31 (12)	11 (8)	10 (8)	23 (23)	19 (28)	
Frequency of physical activity: N (%)													
Less than once a week	16 (9)	17 (12)	59 (17)	30 (27)	2 (4)	<0.001 (0.292)	12 (9)	38 (15)	18 (13)	15 (12)	24 (24)	17 (25)	<0.001 (0.210)
1–2 times a week	60 (36)	52 (35)	131 (39)	49 (44)	8 (16)		52 (39)	95 (38)	65 (46)	13 (10)	45 (46)	30 (44)	
3–4 times a week	69 (41)	61 (41)	128 (38)	24 (22)	3 (6)		52 (39)	97 (38)	38 (27)	55 (44)	26 (27)	17 (25)	
5–6 times a week	21 (12)	12 (8)	15 (4)	3 (3)	13 (25)		16 (12)	16 (6)	14 (10)	15 (12)	1 (1)	2 (3)	
Everyday	3 (2)	6 (4)	6 (2)	4 (4)	25 (49)		1 (1)	7 (3)	5 (4)	27 (22)	2 (2)	2 (3)	
Do you smoke cigarettes (other forms of tobacco, e-cigarettes, etc.) and if so, for how long: N (%)													
Do not smoke	126 (74)	107 (72)	269 (79)	87 (79)	48 (94)	<0.001 (0.185)	97 (73)	187 (74)	104 (74)	115 (92)	80 (82)	54 (79)	<0.001 (0.154)
<5 years	36 (21)	31 (21)	13 (4)	1 (1)	0 (0)		30 (22)	33 (13)	6 (4)	2 (2)	8 (8)	2 (3)	
6–10 years	6 (4)	8 (5)	17 (5)	4 (4)	0 (0)		5 (4)	10 (4)	11 (8)	5 (4)	1 (1)	3 (4)	
11–15 years	1 (1)	1 (1)	17 (5)	6 (5)	0 (0)		1 (1)	8 (3)	9 (6)	2 (2)	2 (2)	3 (4)	
>15 years	0 (0)	1 (1)	23 (7)	12 (11)	3 (6)		0 (0)	15 (6)	10 (7)	1 (1)	7 (7)	6 (9)	
How long do you sleep on average? N (%)													
<6 h	18 (11)	18 (12)	32 (10)	12 (11)	1 (2)	<0.001 (0.231)	16 (12)	27 (11)	10 (7)	5 (4)	4 (14)	9 (13)	<0.001 (0.294)
6 h	51 (30)	27 (18)	75 (22)	33 (30)	2 (4)		41 (31)	58 (23)	39 (28)	8 (6)	26 (27)	16 (24)	
7 h	75 (44)	64 (43)	128 (38)	44 (40)	5 (10)		58 (44)	109 (43)	55 (39)	25 (20)	40 (41)	29 (43)	
8 h	20 (12)	34 (23)	45 (13)	15 (13)	14 (27)		13 (10)	50 (20)	25 (18)	12 (10)	15 (15)	13 (19)	
9 h	2 (1)	4 (3)	54 (16)	3 (3)	28 (55)		2 (1)	5 (2)	8 (6)	73 (58)	2 (2)	1 (1)	
>9 h	3 (2)	1 (1)	5 (1)	3 (3)	1 (2)		3 (2)	4 (1)	3 (2)	2 (2)	1 (1)	0 (0)	
Are you satisfied with your health? N (%)													
Very dissatisfied	2 (1)	0 (0)	8 (2)	0 (0)	0 (0)	<0.001 (0.177)	2 (1)	2 (1)	1 (1)	0 (0)	4 (4)	1 (1)	<0.001 (0.133)
Dissatisfied	22 (13)	16 (11)	36 (11)	15 (14)	3 (6)		18 (14)	22 (9)	18 (13)	3 (2)	15 (15)	16 (24)	
I do not know	22 (13)	18 (12)	50 (15)	26 (24)	3 (6)		21 (16)	45 (18)	15 (11)	13 (10)	16 (16)	9 (13)	
Satisfied	112 (66)	88 (59)	220 (65)	61 (56)	20 (39)		83 (62)	148 (58)	91 (65)	83 (66)	60 (61)	36 (53)	
Very satisfied	11 (7)	26 (18)	25 (7)	8 (7)	25 (49)		9 (7)	36 (14)	15 (11)	26 (21)	3 (3)	6 (9)	

It was also noted that with increasing length of service, the frequency of declared physical activity and its intensity decreased (*p* < 0.001), while the frequency of sleep ≥9 h per day increased, which may be a compensatory response of the body to long-term workload.

## Discussion

The objective of our study was to examine the personal health practices (nutrition, hydration, supplementation, sleep, physical activity) of people in a profession with numerous health risks. According to many studies, lifestyle can affect work safety or increase the risk of injury. Given the physically and mentally demanding nature of firefighters’ work, lifestyle can contribute to work-related health risks (fitness, precision, concentration, resilience, strength, senses).

The health of firefighters is an interesting area of research. Numerous studies describe the monitoring of physiological stress in firefighters using heart rate variability (HRV), a globally recognised parameter for monitoring the activity of the autonomic nervous system (ANS). In these studies, HRV was correlated with BMI, among other things, which is partly consistent with our own study, which also analysed BMI (34, 35).

Health attitudes among firefighters have been widely studied. According to Ras, more than half of firefighters lack adequate knowledge of healthy lifestyle principles, at 52.8 percent of the study population (36). Other analyses indicate that many firefighters are unaware of the link between abnormal body weight or lifestyle and the risk of cardiovascular disease (37, 38). Reports also suggest that firefighters often do not perceive the need to change their lifestyle despite having relevant knowledge (39).

In our own study, we decided to use a proprietary questionnaire. The literature on the subject includes validated tools in the form of questionnaires, protocols for assessing health, occupational stress, or burnout. All preventive measures and scientific research in the firefighter population are an additional source of monitoring the health of firefighters (40–42).

Magnusson notes that firefighters are exposed to repetitive similar experiences (such as participating in firefighting), and over time they may pay less attention or be less cautious about exposure to toxic substances. This puts them at risk of gradually returning to bad habits and routine behaviors that lead to illness or accidents (43). Several studies indicate that firefighters misperceive their own body weight. They underestimate its significance and the degree of overweight or obesity (44, 45).

Body weight and height measurements of SFS firefighters were conducted in Poland in previous years. Wiśniewski estimated that 60% of officers had an abnormal body weight and 10% met the criteria for obesity, although this study was based on a small sample (*n* = 178) (46).

Kraemer notes that professional athletes and participants in strongman competitions can be expected to have a BMI in the range of 43.5 ± 4.8 with a normal to slightly elevated body fat fraction of 18.7 ± 6.2% (47). In the context of firefighters’ health, this phenomenon may explain at least some cases of overweight or even obesity diagnosed solely on the basis of BMI, as is the case, for example, among US Marine Corps soldiers, who often have a BMI in the range of 30–37 with normal body fat levels (48). However, differentiating these cases from actual overweight/obesity is not possible with the accepted research method and requires further analysis. There are also known cases where the BMI did not indicate overweight or obesity in individuals who were diagnosed with these conditions based on other criteria, including BF (49). Nevertheless, BMI appears to have some utility in the aggregate assessment of specific populations. Dawes argues that in police officers, elevated BMI is significantly negatively correlated with performance on physical fitness tests and training (50).

Gaździńska notes that soldiers with higher education and living in large cities score significantly higher on the Health Behavior Inventory questionnaire, which covers nutrition, prevention and health-promoting behaviors, than soldiers with primary education and living outside cities (51). This is consistent with the results obtained, according to which firefighters serving in command positions smoke less frequently and generally report higher levels of satisfaction with their health.

Beyond individual factors, which are the main focus of our study, institutional and social determinants also have a meaningful impact on physical and mental health (workload, institutional policy, workplace ergonomics, organizational culture). Given the scope of our analysis, the structure of the questionnaire, and the predefined hypotheses, we did not explore this area in depth. However, Polish studies (52–54) and international reports (55–57) indicate that socio-institutional factors are linked to firefighters’ health.

### Limitations

The study has several limitations that should be considered. First, its cross-sectional nature prevents causal conclusions. Temporal ordering cannot be established and residual confounding may persist despite stratified analyses. Second, the use of a self-report questionnaire may be associated with self-report errors and response bias. This includes recall and social-desirability bias; moreover, self-reported height and weight can misclassify BMI- particularly in muscular personnel. The lack of objective health measures, such as anthropometric data or clinical biomarkers, limits the accuracy of some indicators. Objective assessments of sleep and physical activity (e.g., actigraphy) and standardized dietary records/biomarkers were also not collected, further constraining measurement precision. Furthermore, the sample was not random, which may affect the generalizability of the results. Volunteer, online recruitment may introduce self-selection and unit-level clustering effects, limiting external validity. Finally, psychological variables, such as stress or sleep quality, which can significantly influence health behaviors, were not assessed. In addition, the proprietary questionnaire did not undergo formal psychometric validation (content/construct validity and internal consistency), which narrows the interpretability of domain scores; any behavioral groupings should therefore be viewed as hypothesis-generating. Future studies should incorporate objective measures, validated psychological scales, probabilistic sampling, longitudinal designs, and formal validation of the instrument.

## Conclusion

This nationwide survey identified clear patterns in firefighters’ health behaviors. Higher BMI was associated with lower participation and frequency of physical activity, and with less favorable diet/sleep patterns (no specific diet, irregular meals, shorter sleep). With increasing service tenure, adherence to customary diet decreased, physical activity declined, and reports of ≥9 h sleep became more frequent (which may suggest compensatory recovery). By role, command staff more often preferred frying and reported lower fluid intake, yet smoked least, used vitamin D more frequently, and rated their health highest. Overweight and obesity were common in the sample, underscoring the need for targeted prevention across career stages. Programs should integrate nutrition, hydration, sleep hygiene, and physical activity, supported organizationally (scheduling, healthy food/water access, protected training time). Future studies should incorporate objective measures, validated psychosocial scales, and longitudinal designs to confirm directionality and assess interventions.

## Data Availability

The raw data supporting the conclusions of this article will be made available by the authors, without undue reservation.
